# Antibiotics and Liver Cirrhosis: What the Physicians Need to Know

**DOI:** 10.3390/antibiotics11010031

**Published:** 2021-12-28

**Authors:** Caterina Zoratti, Rita Moretti, Lisa Rebuzzi, Irma Valeria Albergati, Antonietta Di Somma, Giuliana Decorti, Stefano Di Bella, Lory Saveria Crocè, Mauro Giuffrè

**Affiliations:** 1 Department of Medical, Surgical and Health Sciences, University of Trieste, 34127 Trieste, Italy; caterina.zoratti95@gmail.com (C.Z.); moretti@units.it (R.M.); lisa.rebuzzi@gmail.com (L.R.); i.albergati91@gmail.com (I.V.A.); antonietta.disomma93@gmail.com (A.D.S.); stefano932@gmail.com (S.D.B.); lcroce@units.it (L.S.C.); 2 Institute for Maternal and Child Health-IRCCS Burlo Garofolo, 34137 Trieste, Italy; decorti@units.it; 3 Italian Liver Foundation, 34149 Trieste, Italy

**Keywords:** antibiotics, liver cirrhosis, ascites, spontaneous bacterial peritonitis, hepatic encephalopathy

## Abstract

The liver is the primary site of drug metabolism, which can be altered by a variety of diseases affecting the liver parenchyma, especially in patients with liver cirrhosis. The use of antibiotics in patients with cirrhosis is usually a matter of concern for physicians, given the lack of practical knowledge for drug choice and eventual dose adjustments in several clinical scenarios. The aim of the current narrative review is to report, as broadly as possible, basic, and practical knowledge that any physician should have when approaching a patient with liver cirrhosis and an ongoing infection to efficiently choose the best antibiotic therapy.

## 1. Introduction

The liver is the primary site of drug metabolism. Biotransformation of drugs and xenobiotics into more hydrophilic compounds is necessary for their elimination through the urine and the bile. Most drugs are metabolized by phase I (mainly oxidation, reduction, and hydrolysis, leading to the introduction of functional groups and often changing the biological properties) and phase II (that involves conjugation with an endogenous substance such as glucuronic acid, sulfate, glycine) reactions. These steps are largely dependent on two factors: hepatic blood flow and the metabolic capacity of the liver [1].

Liver cirrhosis is the final common pathological pathway of liver damage arising from many chronic liver diseases. Although there are several causes of liver cirrhosis, some histopathological changes in the liver parenchyma, shared between the various etiologies, include degeneration and necrosis of hepatocytes and the replacement of liver parenchyma by fibrotic tissues, regenerative nodules, and loss of liver function [2].

**Figure 1:**
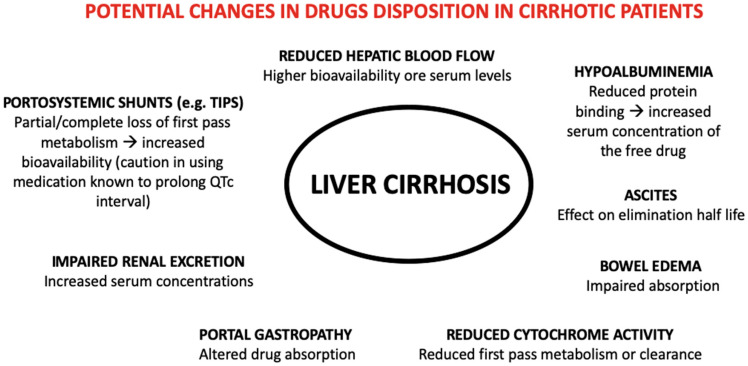
Potential changes in drug disposition in cirrhotic patients.

In liver cirrhosis, impaired drug disposition is due to many pathological changes, including liver cell necrosis, portosystemic shunt, reduction in the concentration of drug-binding proteins, atypical drug volume of distribution, altered drug metabolism and elimination, altered pharmacodynamics, drug interactions, and the frequent association with renal failure [1] as shown in Figure 1. In particular, the impairment of drug metabolism is directly related to the degree of liver dysfunction and, although various tests can be used to predict the severity of liver function (e.g., indocyanine green clearance, Child Pugh or Meld-Na [1].

Of notice, the liver metabolic capacity must be reduced more than 90% to actually require dosage adjustment. However, the use of medications in patients with cirrhosis is usually a matter of concern for physicians, considering the risk of developing an acute drug-induced liver injury (DILI) or hepatic encephalopathy (HE). Furthermore, worsening of renal failure and inducing gastrointestinal bleeding are likely scenarios in these patients.

The presence of ascites and large volume paracentesis can affect the volume of distribution (VD) of many drugs, and as a result, elimination half-life may be affected [3,4]. Coagulation disorders, the effect of transjugular intrahepatic portosystemic shunts (TIPS), or other factors on hepatic blood flow are unique settings that can alter drug distribution, metabolism, and excretion. Therefore, it is essential for physicians to be able to determine how to efficiently prescribe antibiotics that are used to treat both hepatic and non-hepatic-related disorders in this population [5]. Appropriate antibiotic therapy selection and dose adjustment can contribute to optimal clinical outcomes while decreasing the risk of mortality and hepatotoxicity.

### 1.1. Hypoalbuminemia

Albumin is the most abundant plasma protein in humans, and is produced by liver cells, with synthesis rates around 200 mg/kg body weight/day [6,7,8]. Approximately 40% of albumin is found in the vascular system and 60% in the extravascular space [6]. The half-life is about 21 days, with 4% daily degradation rates [6,9].

Hypoalbuminemia is defined by serum albumin <35 g/L, although clinically significant hypoalbuminemia is identified by levels <25 g/L [6]. The main pathogenetic mechanisms are reduced synthesis (e.g., cirrhosis, acute and chronic hepatitis, and malnutrition), increased catabolism (e.g., infections and cancer), alteration in distribution (e.g., fluid loss or surgery), and increased loss through kidney, skin, and bowel [10].

Hypoalbuminemia frequently occurs in cirrhotic patients due to impaired hepatocyte synthesis, increased sodium and water retention, and greater transcapillary escape rate. Albumin may be therapeutic in cirrhotic patients, especially as plasma expanders after a large volume paracentesis, spontaneous bacterial peritonitis, and type 1 hepatorenal Albumin plays a pivotal role in the maintenance of homeostasis. Among its multiple roles, this protein can bind to different substances, such as drugs. Consequently, low serum albumin is associated with alterations in the binding of many drugs that have a high (>90%) bound profile. Among currently used antibiotics, ceftriaxone is 83–96% bound to albumin, flucloxacillin 95%, cephalothin 55–75%, aztreonam 60% [10], the carbapenems ertapenem (85–95%) and faropenem (96–99%) are both highly albumin-bound [10]. Considering glycopeptides, vancomycin is bound to albumin between 30% and 60%, while teicoplanin is 90–95% [10]. A study compared the effectiveness of ertapenem, with other carbapenems, such as meropenem or imipenem, in the presence of hypoalbuminemia against infections sustained by carbapenems-susceptible *Enterobacteriaceae* in hospitalized patients [11].

The results showed a higher 30-day mortality incidence with ertapenem compared to imipenem/meropenem [11]. Moreover, using the regression models, it could be inferred a five-fold mortality risk for a subject treated with ertapenem and an albumin level of 2 g/dL compared to a subject with normal albumin (4 g/dL), whereas there was no such association among subjects treated with imipenem or meropenem [11].

For highly protein-bound antibacterials, hypoalbuminemia increases the unbound fraction of the administered drug, which is available for distribution and clearance from the plasma. Only the unbound drug can distribute into body tissues, exerting its pharmacological effect and potential toxicity, and be eliminated from the vascular compartment. Hence hypoalbuminemia increases the apparent total VD and the clearance (CL) of the drug, decreasing the effective concentration in serum over time, which could be a problem when facing bacteria with high minimal inhibitory concentration (MIC). Besides taking into consideration renal impairment and hepatic insufficiency in drug dose adjustments, hypoalbuminemia should also be considered to optimize antibiotic treatment [10].

Moreover, the percentage of antibiotics bound by albumin may also be altered by other clinical scenarios that can occur in cirrhotic patients, such as increased concentration of endogenous molecules with high affinity for albumin (e.g., in uremia), increased free fatty acids, or bilirubin levels or administration of other highly protein-bound drugs can displace highly bound antibiotics from their albumin binding sites. The pharmacokinetic alterations caused by hypoalbuminemia could have significant consequences on the attainment of pharmacokinetic/pharmacodynamic targets. The higher VD could be a problem for concentration-dependent drugs, such as the aminoglycosides, requiring a higher loading dose to reach therapeutic levels. On the other hand, faster CL may affect the efficacy of ß-lactams. Penicillins, cephalosporins, carbapenems, and monobactams belong to the ß-lactams family and are time-dependent antibiotics. Their effect depends on the percentage of time in which serum concentrations of the free drug remain above the MIC of the causative pathogen (% fT > MIC). For maximal bactericidal activity, penicillins and monobactams require at least 50–60% fT > MIC [10], cephalosporines require 60–70%, and carbapenems 40% fT > MIC [10].

Higher CL and VD due to hypoalbuminemia lead to low drug concentrations, especially for hydrophilic drugs, which may be below fT > MIC targets and thereby ineffective. Therefore, to maintain free drug levels above the MIC, optimizing their pharmacokinetic/pharmacodynamic target, also given the short half-life of most ß-lactams, continuous or extended infusions of these agents may be used, and have shown to improve 30-days survival in patients with cirrhosis [12].

In conclusion, the outcome of antibacterial therapy depends on achieving therapeutic concentrations of unbound antibacterial at the target site of infection. Empirical dosing regimens are usually derived from healthy study populations. Therefore, further studies should be mandatory to optimize antibacterial dosing in hypoalbuminemic patients, mainly when infections are sustained by pathogens with high MIC. In daily clinical practice, increased doses of antibiotics could be necessary for achieving the optimal antibacterial target. Loading doses for at least the first 24 h of treatment should then be considered. In their review, Ulldemolins et al., suggest prescribing doses 50–100% higher than standard doses as a loading dose for highly bound drugs in moderate to severe hypoalbuminemia cases in critically ill patients with moderate to severe hypoalbuminemia. Dose adjustment must be driven by the predicted CL, considering albumin levels and other factors influencing CL (e.g., renal function, hepatic function.) [10]. Strict monitoring of blood levels for antibiotics with a low therapeutic index could also be helpful.

### 1.2. Infections and Liver Cirrhosis

Patients with cirrhosis are at increased risk for bacterial infections, one of the most common complications and a significant cause of death in these patients [13], with an overall incidence ranging between 25% and 40% in patients with decompensated cirrhosis, about five times higher than in the general population [14].

Patients with cirrhosis are more susceptible to infections because they are both in a state of immune dysfunction and of excessive activation of proinflammatory cytokines. The immune dysfunction leads to an altered defense against bacterial agents, associated with reduced bacterial clearance, facilitating gut bacterial overgrowth and translocation induced by increased intestinal permeability [15]. On the other hand, the enormous activation of proinflammatory cytokines and the increase in the circulating levels of tumor necrosis factor (TNF)-alpha and interleukin (IL)-6 contribute to sepsis-related organ failure. This pro-inflammatory phase is followed by a prolonged “immune-paralysis”, known as compensatory anti-inflammatory syndrome (CARS), which facilitates secondary infections and death [16]. Cumulative infection-related mortality for cirrhotic patients is 43.5% [17], with cirrhotic patients having a 2-fold risk for infection-related death if compared to non-cirrhotic patients, especially taking into consideration the 70% mortality risk in cirrhotics with septic shock [18]. The main risk factors associated with increased infection prevalence are severe liver impairment and consequences of clinically significant portal hypertension.

Recent evidence also notes that patients with non-alcoholic fatty liver disease (NAFLD) show propensity for infection, even in the absence of overt cirrhosis [19]. This is probably due to various mechanism such as the ongoing low grade of inflammatory status, the deficient function of both hepatic natural killer cells and neutrophils in the contest of insulin resistance [20], vitamin D deficiency [21,22], and an increased intestinal permeability as a consequence of both bowel intestinal overgrowth and tight junction alterations in the small bowel epithelium [23,24]. Although there are a large number of theories, the exact pathological reason by which patients with NAFLD are more susceptible to infections remains unclear.

The most common type of infections in cirrhotic patients involve spontaneous bacterial peritonitis (SPB) in 25–30% of cases, followed by urinary tract infection (UTI) (20–25%), pneumonia (15–20%), and soft tissue infection (10%) [25,26,27].

In about 75% of cases, infections are diagnosed at hospital admission and are treated as community-acquired infections, while in the remaining 25% of cases patients get infected during the hospitalizations [28,29,30].

Bacteria involved in community-acquired infections among patients with liver diseases are susceptible to commonly employed antibiotics. In the nosocomial setting, the extensive use of broad-spectrum antibiotics has facilitated the spread of multi-drug resistant (MDR) bacteria, among which the most common are extended-spectrum beta-lactamase (EBLS)-producing *Enterobacteriaceae*, followed by non-fermentative Gram-negative bacilli (i.e., *Pdeudomonas aeruginosa*, *Stenotrophomonas maltophilia*, and *Acinetobacter baumanii*); carbapenemase-producing *Enterobacteriaceae*; methicillin-resistant *Staphylococcus aureus* (MRSA) and vancomycin-susceptible or resistant enterococci (VSE, VRE) [23,31,32]. Consequently, the efficacy of empirical antibiotic treatment is decreased in nosocomial infections compared to community-acquired and healthcare-associated episodes [31]. Infections caused by MDR bacteria are becoming a big challenge to face, considering that they negatively impact short-term prognosis, increasing treatment failure, septic shock, and hospital mortality [23,31,32]. It is foreseeable that infections caused by multi-resistant microorganisms will become more common and challenging to manage in the near future. In developing countries urinary UTIs caused by MDR-bacteria are becoming frequent in hospital settings and they represent a significant factor of mortality among patients with liver cirrhosis [33]. UTIs can clinically occur in different forms, from the uncomplicated cystitis to the severe pyelonephritis than can lead to septic shock, therefore it is important to prevent these infections, to know how to recognize them promptly and to undertake the correct antibiotic strategy [33].

The epidemiological pattern of MDR bacteria differs among geographical areas, probably due to different prescription policies of antibiotics in different countries, and regular assessment of local epidemiology is recommended.

It is, therefore, crucial to establish an appropriate therapy in patients with cirrhosis and bacterial infection.

## 2. Ascites and Spontaneous Bacterial Peritonitis

Ascites is the most common complication of liver cirrhosis, with 60% of compensated patients developing ascites within ten years from cirrhosis diagnosis [34]. Ascites develop and worsen in parallel to the building up of portal hypertension, and it is associated with poor prognosis and a poor quality of life [35,36].

SBP is defined as the infection of a previously sterile ascitic fluid without any clear intra-abdominal infective origin. In 40–60% of SBP cases, the responsible microorganism can be identified from ascitic fluid/blood cultures [26,27,35,37,38]. SBP often presents with abdominal pain and fever, eventually followed by gastrointestinal manifestation (nausea, vomit, and diarrhea), hepatic encephalopathy, and renal impairment. SBP diagnosis is based on the detection of an ascitic fluid polymorphonuclear (PMN) count ≥250 cells/mm^3^, which also represents a recommendation to empirically start antibiotic treatment [37,39,40]. SBP overall mortality may exceed 90%, however early diagnosis and treatment can drastically reduce the risk to approximately 20% [35,41]. SBP pathogenesis appears to be related to decreased bacterial clearance associated with structural and functional alterations in intestinal mucosa that often develops in cirrhotic patients [42,43,44,45]. Regardless of the pathophysiology behind SBP development, the most common isolated bacteria in SBP are *Escherichia coli* and Gram-positive cocci (i.e., streptococci and enterococci).

The following paragraphs will report the current treatment regimens/prophylaxis strategy for SPB in cirrhotic patients (also summarized in Table 1).

### 2.1. Empirical Antibiotic Therapy and Prophylaxis of Community Acquired PBS

Empirical antibiotic therapy should be prescribed immediately after the diagnosis of SBP, while ascitic fluid culture results are still pending [37,38]. For example, cefotaxime, a third-generation cephalosporin, has been broadly studied in SBP therapy considering that it covers most causative organisms and reaches high ascitic fluid concentrations during administration [46,47]. A 2 g dose of cefotaxime every eight hours has been shown to produce an excellent concentration in the ascitic fluid and clinical effectiveness [48]. Alternatively, a 2 g dose of ceftriaxone per day [49] has been shown to prevent SPB in cirrhotic patients with gastrointestinal hemorrhage [50].

Furthermore, amoxicillin-clavulanate, first given intravenously and then orally, has shown to have similar results on SBP resolution and mortality compared with cefotaxime [47] and could be a valid alternative to cefotaxime for the empirical treatment of bacterial infections in cirrhosis.

Ciprofloxacin can be used for patients who cannot take a cephalosporin (e.g., allergic patients), although it does not reach the ascitic fluid as well as cefotaxime [51]. In general, ciprofloxacin is given at a dose of 400 mg intravenously twice daily to patients with normal renal function.

Specific oral agents may be as effective as parenteral therapy in treating uncomplicated SBP [51,52]. A trial demonstrated comparable outcomes with a short course of intravenous ciprofloxacin (200 mg every 12 h for two days) followed by oral ciprofloxacin therapy (500 mg every 12 h for five days) compared with intravenous therapy alone for seven days [51]. Oral ofloxacin 400 mg twice daily has given similar results as intravenous cefotaxime in uncomplicated SBP, which means without renal failure, hepatic encephalopathy, gastrointestinal bleeding, ileus, or shock [52].

Primary prophylaxis should be considered in patients with cirrhosis at high risk for SBP (i.e., low serum albumin and severe liver/renal dysfunction). Moreover, in all patients with a prior episode of SBP, it is crucial to begin long-term prophylaxis. In terms of secondary prophylaxis data shows that norfloxacin (400 mg/day) could reduce SBP recurrence from 70% to 20% [53] In these settings, secondary prophylaxis should be introduced immediately after the first episode of acute SBP and should be continued until liver transplantation or the resolution of ascites [17].

**Cefotaxime**. Among third-generation cephalosporins, cefotaxime is the first-line therapy in community-acquired infections. The liver metabolizes about 40–50% of cefotaxime to an active deacetylated metabolite [54]. Two pharmacokinetic studies in cirrhotic patients demonstrated a threefold increase in cefotaxime’s half-life in comparison to healthy subjects, while one study did not find any significant difference [55,56,57]. However, due to the very high therapeutic index of the antibiotic, despite these pharmacokinetic alterations, dose adjustment is not necessary for patients with hepatic impairment [55].

**Amoxicillin-clavulanate**. Amoxicillin-clavulanate is eliminated via renal tubular secretion and does not require dose adjustment in cirrhotic patients.

**Ciprofloxacin and Norfloxacin**. All fluoroquinolones are poorly bound to proteins. Norfloxacin and ciprofloxacin are eliminated both by the kidney and the liver. Hepatic biotransformation leads to several metabolites which partially maintain an antibacterial activity [58]. Studies suggest that, in patients with cirrhosis, there is no need to adjust ciprofloxacin doses [3,4]. Although no pharmacokinetic study in patients with hepatic dysfunction has been published for norfloxacin, the drug has been largely employed in the clinics, with excellent tolerability [53].

**Trimethoprim/sulfamethoxazole (TMP/SMX)**. Trimethoprim/sulfamethoxazole blocks two consecutive steps in the biosynthesis of nucleic acids and proteins that are essential for the persistence of many bacteria in the organism [59]. Two studies compared the effectiveness of trimethoprim/sulfamethoxazole versus norfloxacin in the prophylaxis of SBP in cirrhotic patients [60,61]. The results in both studies showed that trimethoprim/sulfamethoxazole and norfloxacin are equally effective in SPB primary and secondary prophylaxis, however patients treated with TMP/SMX developed more frequently adverse effects [60]. On the other hand, TMP/SMX is an economic drug [62,63] and it is easily available in public health system in many countries, therefore TMP/SMX could be considered a valid alternative to norfloxacin in the contest of SPB prophylaxis.

### 2.2. Nosocomial SBP Therapy

Nosocomial bacterial infections are a relevant complication in hospitalized [58,64]. In particular, nosocomial SBP (NSBP) may arise in up to one-third of hospitalized cirrhotics with an overall 30-days survival which does not reach 20%, especially in those patients where empirical treatments are not started promptly [65]. The most frequently isolated MDR bacteria in NSBP are represented by ESBL-producing *Enterobacteriaceae*, non-fermentable Gram-negative bacilli (such as *P. aeruginosa*), MRSA, and VRE [66]. Unfortunately, current guidelines for treating SBP do not provide a helpful distinction between community-acquired and nosocomial episodes [65].

Although a 2013 position paper recommended piperacillin/tazobactam or meropenem with daptomycin for NSBP [23], also confirmed by some field experts [67,68] who also suggested the use tigecycline to cover ESBL- producing *Enterobacteriaceae* [31].

Piperacillin/tazobactam as the risk to be inadequate for patients with life-threatening infections due to ESBL-producing *Enterobacteriaceae* [69].

**Meropenem** has excellent bactericidal activity against ESBL-producing *Enterobacteriaceae*, whereas it is poorly active against staphylococci and enterococci [70].

**Glycopeptides** (e.g., vancomycin, teicoplanin) should be used carefully because of the potential risk of nephrotoxicity. In particular, the rate of acute kidney injury is higher in patients with concomitant acute liver failure, which may be related to hemodynamic instability or the development of hepatorenal syndrome [71].

**Daptomycin** is a lipopeptide active against MDR Gram-positive pathogens, including methicillin-susceptible *S. aureus*, MRSA and VRE [72]. However, there is a decreased susceptibility to daptomycin (DAPR) reported in MRSA [73]. Despite this observation, the combination of daptomycin/beta-lactams has been proven clinically effective for preventing and treating infections due to DAPR-MRSA strains [74].

**Tigecycline**. Tigecycline pharmacokinetic properties were assessed in patients with different stages of liver cirrhosis [64], with no significant difference in the pharmacokinetics between patients with Child-Pugh class A or B and healthy controls. Nevertheless, patients in Child-Pugh class C showed half of the clearance compared with controls [64]. Thus, implying that patients with mild to moderate liver cirrhosis do not require dose adjustments, whereas patients with more severe disease should have tigecycline maintenance doses reduced by 50%.

In conclusion, different therapeutic options are recommended according to the onset of the infection, third-generation cephalosporins are preferred for community-acquired infections, but they do not have adequate microbial coverage for treatment of nosocomial SBP. Cirrhotic patients with nosocomial SBP in a clinical setting with a high prevalence of VRE, MRSA, ESBL should receive as empirical antibiotic therapy: high dose of daptomycin (i.e., 8–12 mg/kg every 24 h) plus meropenem (i.e., 1 g/8 h) [65,75].

## 3. Hepatic Encephalopathy

HE is defined as “brain dysfunction caused by liver impairment and/or portosystemic shunting manifesting as a wide spectrum of neurological or psychiatric abnormalities ranging from subclinical alterations to coma” [76].

This pathological condition is a prevalent complication of portal hypertension and cirrhosis, occurring in 50–70% of patients. Considering signs and symptoms of HE, the clinical spectrum may be highly variable and better defined as a continuum. It affects patients’ behavior, cognitive sphere, and motor skills. There is an inevitable heterogeneity not only between different patients but also longitudinally for an individual patient. This makes HE a not always so easy condition to diagnose and treat. According to ISHEN Classification [77], HE can be classified as in Overt (OHE), clinically evident with disorientation and asterixis as the most specific signs; and Covert (CHE), not clinically evident but demonstrable by psychometric and electrophysiological tests. At this time, only OHE is routinely treated, whereas Minimal HE and CHE, which are not easy to define, have no clear treatment recommendation [78].

Most liver-failure patients develop minimal hepatic encephalopathy (MHE). MHE can progress to clinical HE, which can evolve to coma and death.

There is a large variability in the onset of MHE and the progression to clinical HE.

Most patients remain undiagnosed. Patients may survive for years with progressive deterioration of neurological function. Nevertheless, MHE reduces the ability to perform executive functions in daily living, diminishes the quality of life, predisposes to clinical HE, and reduces lifespan. HE is characterized by motor slowness that ends in dysmetric movements, progressive cognitive and psychiatric alterations, cerebral edema, and coma [79,80,81]. The urgency of new diagnostic criteria is a strong need because it represents a molecular tsunami inside the brain whenever HE is overcoming.

The two main pathogenic factors traditionally considered for HE are hyperammonemia and inflammation. Major corrections inside these two historical factors have been recently demonstrated. The effects of hyperammonemia on the brain depend on several factors: the concentration of ammonia; the speed at which the levels of ammonia increase; the duration of hyperammonemia; the period of brain development in which HE occurs. When animal models are subjected to acute ammonia intoxication, the concentration of ammonia in the brain rapidly increases from a basal level of approximately 0.2 μmol g^−1^ to 1–3 μmol g^−1^. Regardless of ammonia concentration, the brain’s only way for its removal is the astrocytic glutamine synthesis, presented by spectroscopic measures [82,83] inside HE brains.

In rats, acute liver failure involves selective alterations of specific brain areas (i.e., the cerebellum), with substantial blood-brain barrier leakages, promoting the first steps of vasogenic edema. Hyperammonemia leads to hyperactivation of N-methyl D-aspartate (NMDA) receptors. They activate the glutamate–nitric oxide–cyclic GMP pathway, producing excessive nitric oxide and cGMP [84]. Both the facts contribute to the animal’s death by excitotoxic mechanisms. The astrocytic glutamine accumulation induces water symport, which might cause astrocytic swelling and cytotoxic edema [85]. At a later stage, NMDA receptor activation increases lactate and CBF alterations (increase in the cortex and decrease in the cerebellum), further increasing ICP, contributing to death [86].

Hyperammonemia per se induces neuroinflammation. In cultured microglia, ammonia upregulates the microglial activation marker (allograft inflammatory factor 1), elevated in the HE-human brain [87]. Acute liver failure promotes regional neuroinflammation, associated with a consequent modification of cerebral blood flow, more evident in the brain cortex but not in the cerebellum [87].

On the contrary, animal models of chronic HE show cerebellar inflammation, with a concomitant increase of tumor necrosis factor and an intracranial elevation of Il-2, Il-6, and Il-8 [88]. Moreover, microglial activation is an overwhelming mechanism because its activation is not only an intrinsic one but might be regulated by the blood-brain barrier leakages, through which pro-inflammatory cytokines can pass, or by direct infiltration of blood-immune cells. Blood cytokines may also stimulate receptors on endothelial cells and trigger the release of inflammatory factors into the brain [89].

The traditional role of brain edema in HE is under debate, with recent functional MRI (fMRI) studies. The conventional vision of the vasogenic edema as the beginner of the neurological alterations is defeated by animal models of HE undergoing fMRI. They show an unexpected increase in the apparent diffusion coefficient (just the opposite of what is commonly detected in cytotoxic edema). They offer motor and executive alterations in the absence of brain edema a [90]. Magnetoencephalography studies evidenced an altered synchronization of the thalamocortical coupling in HE animal and human HE models [91,92]. Resting-state fMRI studies show that MHE patients have a selective default-mode thalamus-basal ganglia-frontal cortex, with substantial decreased functional connection alterations [93]. Therefore, brain edema is considered just a final part of the sequence of events that occur in the HE brain.

The most essential accepted pathogenic mechanism of HE is the severe impairment of neurotransmitters exerted by ammonia. Independently by its concentrations, ammonia inhibits postsynaptic chloride extrusion [94] and the postsynaptic effect of glutamate receptors [95]. Rat hippocampal models showed inhibition of glutamate uptake capacity by blood samples of human HE patients, and the inhibition is directly related to the ammonia concentrations [96]. Moreover, ammonia inhibited the high-affinity glutamate uptake into synaptosomes of rat astrocytes [97]. This situation is furthermore confirmed by the reveal of a significant loss of glutamate transporter (GLT-1, dispersed all around the brain and GLAST, localized in the cerebellum) in astrocytes and (EAAC-1) in neurons of cerebellar rat sections [98]. The drastic reduction of GLT-1 and GLAST expression determined an increase of extracellular glutamate release, with a consequent downregulation of the AMPA/kainate receptors, as a rapid response to acute liver failure; chronic liver failure directly damaged NMDA neural receptors [99,100]. It has even been suggested that the demonstrated decrease of AMPA/kainate receptors implies an overt hyper-expression of NMDA receptors in acute phases, which might benefit from the memantine supplementation [101,102,103,104]. Finally, it has been argued that acute NMDA hyper-expression can determine a possible downregulation of dopamine inside the basal ganglia [105]. It has been proposed that ammonia modifies GABA agonists to their GABA-A ligands, defining the so-called barbiturate–like action of HE, and definitively affects GABA uptake [106]. More important is the possible effect of ammonia on GABA different receptor types. Ammonia acts on the post-synaptic GABA-benzodiazepine receptor complex and on the PTBR receptors, which are localized all around the body but are strongly localized on the astrocytic mitochondria. There is a constant increment of their densities all around the astrocytes during HE status, increasing the PTBR mRNA in HE experimental condition. Exposure of cultured glioma cells to PTBR ligands results in astrocyte proliferation and mitochondrial swelling as observed in HE [107,108,109]. The deposition of heavy metals, in particular manganese, inside the globus pallidus in chronic liver failure, contributes to the alterations of astrocytic functions inside the basal ganglia, interrupting several motor refinement networks, leading to some movement alteration observed in chronic liver patients [110,111]. PTBR activation associated with manganese exposure results in increased allopreg-nanolone synthesis, a potent GABA-A receptor agonist [111]. Together with all these alterations, a constant increase of the serotonin metabolite 5-hydroxy indole acetic acid (5-HIAA) has been found in the cerebral spinal fluid of all the chronic liver failure patients, with an increased brain serotonin degradation and a synaptic deficit of it.

The serotonin synaptic deficit determines a constellation of neuropsychiatric symptoms such as altered sleep patterns, depression, and personality changes [112]. On the other hand, homovanillic acid, as dopamine metabolite, is increased in HE, probably due to the accumulation of manganese, as above reported, and to an increment of dopamine oxidation [113]. Histamine is another target for HE; rats models show an increase of histamine up to 6 months post-liver failure, including a loss of histamine in H3 receptor sites [114,115], determining a loss of circadian rhythms, and electrical brain alterations, which might resemble the human hepatic coma [115]. Finally, brain extracts from animal HE models contain altered beta-endorphin levels, and their brains demonstrate region-selective alterations of the mu and delta-opioid receptor sites [116,117].

### Antibiotic Use in Hepatic Encephalopathy

As stated above, multiple components play a role, alone or in combination, in the pathophysiology of HE. It is important to remember that HE is a reversible condition, and the correct treatment positively influences patients’ survival, the possibility of recurrence, and the quality of life. HE treatment varies according to the severity of presentation and the goals of therapy. Most treatments are based on manipulating the intestinal microbiota. Therefore, antibiotics that act on the gut represent a key treatment strategy. It is essential to remember the role of infection as a trigger of HE, the role of gut bacterial metabolism in ammonia generation [118] and the connection between the pro-inflammatory milieu and alterations of intestinal microbiota, which is further worsened by infections [119]. As a matter of fact, the rationale of antibiotic therapy in these patients is preventing the production and absorption of gut-derived neurotoxins (e.g., ammonia) and reducing inflammation [78]. In general, it is preferred to avoid prolonged use of these drugs. Long-term administration can lead to antimicrobial resistance, diarrhea, malabsorption, and systemic effects due to minimal absorption, mostly in damaged gastrointestinal mucosa. Rifaximin, neomycin, paromomycin, metronidazole, and vancomycin are the main studied antibiotics (as summarized in Table 2).

**Rifaximin**. Rifaximin has, so far, the greatest evidence. It showed a broad spectrum activity against Gram-positive, Gram-negative, and anaerobic bacteria, by binding DNA-dependent RNA polymerase and disrupting RNA synthesis. Current guidelines recommended the use of lactulose as the first choice for treatment of episodic HE and prevention of recurrent episodes of HE after the first episode [120]. Rifaximin is an effective add-on therapy to lactulose for the prevention of HE recurrence after the second episode [76,121]. Its use seems to reduce the risk of developing HE and hospitalization and improve health-related quality of life. When coupled, rifaximin and lactulose seem to have better results [121]. In numerous trials, rifaximin, 550 mg orally every 12 h or 400 mg orally every 8 h, has also been demonstrated to be Normal gut flora may be affected by this drug: high doses were shown to decrease *Enterococcus* spp., *E. coli*, *Lactobacillus* spp., *Bacteroides* spp., *Bifidobacterium* spp. and *Clostridium perfringens*, all of which returned to initial values after a wash-out period. Being virtually non-adsorbed by the gastrointestinal tract, rifaximin has minimal side effects: the most common are flatulence, abdominal pain, headaches, constipation, nausea, and vomiting. Furthermore, no reported drug interactions make it relatively safe.

**Aminoglycosides (neomycin and paromomycin)**. Neomycin is effective against most Gram-negative aerobes, except some Pseudomonas strains, and against *S. aureus* and *Enterococcus faecalis*. It inhibits bacterial protein synthesis via binding to the bacterial 30S ribosomal subunit, causing misreading and premature termination of mRNA translation. Neomycin is usually orally administered, 1 g every six hours for up to six days in an acute episode of overt HE and 1–2 g daily for chronic use [78]. Although neomycin is poorly absorbed from the gastrointestinal tract, ototoxicity and nephrotoxicity may occur. Due to these side effects and lack of demonstrated clinical benefit, neomycin clinical use is not recommended.

The use of aminoglycosides should therefore be reserved for patients with severe bacterial infections that cannot be treated with other antibiotics. 

Paromomycin has also been utilized but the evidence for it is limited and therefore it is not approved for the treatment of HE [78,122].

**Metronidazole**. Metronidazole inhibits protein synthesis by interacting with DNA and causing helical DNA structure and strand breakage loss. It acts against anaerobic bacteria, protozoa, and microaerophilic bacteria. Because of its prolonged rate of elimination in HE patients, increased risk for irreversible peripheral neurotoxicity, and limited studies on the efficacy, metronidazole is no longer recommended to manage the acute episode or chronic management of HE [78,123]. Disulfiram-like reactions in patients drinking ethanol or taking ethanol-containing medications may occur too [123].

**Vancomycin**. It inhibits the polymerization of peptidoglycans in bacterial cellular walls. It is prevalently used to treat/prevent bacterial infections caused by Gram-positive bacteria, including MRSA. It is effective against enterococci, streptococci, and methicillin-susceptible *S. aureus* (MSSA) infections. This drug may be safer for managing an acute HE episode, but in the face of an increased prevalence of VRE and other bacterial resistance and limited studies, vancomycin is not recommended [31,78].

## 4. Renal Impairment

In cirrhotic patients, decompensated hepatic failure leads to renal vasoconstriction and subsequent renal failure, which reduces renal elimination of agents with primary renal clearance, leading to increased serum drug concentrations [3,5,124,125]. Nephrotoxicity is one of the most common adverse drug reactions (6.8%), and most adverse drug reactions occur in patients with advanced cirrhosis (Child-Pugh C), often with renal impairment [5]. Another aspect to highlight is that patients with cirrhosis have reduced effective renal plasma flow and glomerular filtration rates, even in the absence of ascites [126,127]. Moreover, patients with liver cirrhosis have low serum creatinine concentrations (due to impaired synthesis of creatinine and reduced muscle mass), which may indicate that actual glomerular filtration rates cannot be estimated from serum creatinine levels [128,129].

Furthermore, cirrhotics have decreased, thus drugs with renal elimination and narrow therapeutic range should be dosed with extreme caution in these patients [126]. Several antibiotics have shown diminished renal elimination, such as in the case of ofloxacin, ampicillin, aminoglycosides, and vancomycin.

**Ofloxacin**. The metabolism is affected by renal dysfunction in patients with ascites. Of notice, ofloxacin penetration into the ascitic fluid is excellent with effective therapeutic concentrations [130,131]. Lower renal excretion in cirrhosis has been proven for ofloxacin, and dose adjustments are warranted [5,126].

**Ampicillin**. For drugs such as ampicillin, pharmacokinetic parameters are unchanged in cirrhosis, but dose reductions are recommended for patients with renal impairment [132]. **Aminoglycosides and vancomycin**. Serum levels of aminoglycosides must be monitored closely in these patients to decrease the risk of renal failure [133]. Intravenous (IV) vancomycin can cause increased toxicity in patients with liver failure [1]. The combination of aminoglycosides and IV vancomycin is generally contraindicated given their relatively high risk of inducing nephrotoxicity and renal failure [1,5,134].

Infection treatment in patients with liver cirrhosis also includes strategies to prevent acute kidney injury (AKI). According to the International Ascites Club and the Acute Dialysis Quality Initiative, AKI in liver cirrhosis is defined by an increase in serum concentration by 0.3 mg/dL in less than 48 h or by a 50% increase from stable baseline concentrations in the previous six months, without taking into consideration the final serum creatinine level [135,136]. AKI usually develops in 27–34% of patients with cirrhosis following infections [25]. Other risk factors for infection-induced AKI in cirrhosis include advanced liver disease [137], preexisting kidney disease hypovolemia, low cardiac [25], unresolved infection [137].

In cirrhotics with SBP, the administration of human albumin drastically reduces the incidence of AKI and improves overall survival [138,139]: in particular, albumin (1.5 g/kg within six hours of SBP diagnosis, followed by 1 g/kg on day 3) plus intravenous antibiotics reduced incidence of renal impairment from 33% to 10% and mortality from 29% to 10% [138]. However, albumin infusion appears to be more effective in those patients with baseline bilirubin ≥ 4 mg/dL, creatinine ≥ 1 mg/dL, or blood urea nitrogen ≥ 30 mg/dL [39]. In addition, to actively prevent AKI, all potentially nephrotoxic drugs, such as non-steroidal anti-inflammatory drugs (NSAIDs), vasodilators, and aminoglycosides, should be avoided [135].

### Hepatorenal Syndrome

As already stated, patients with hepatorenal syndrome are at higher risk of reduced antibiotics renal clearance, thus needing dose reductions [125]. In particular, during infection, an acute kidney injury that does not respond to albumin infusion is considered as hepatorenal syndrome [140]. Infections, such as SBP, can precipitate active liver decompensation manifestations or may promote the occurrence of new decompensating symptoms, and are the major precipitants of acute-on-chronic liver failure (ACLF) in Western countries [14,141,142,143].

## 5. Transjugular Intrahepatic Portosystemic Shunts (TIPS)

Transjugular intrahepatic portosystemic shunts (TIPS) are placed to manage complications from clinically significant portal hypertension [5]. Portosystemic shunting is the leading cause of altered drug metabolism in liver cirrhosis [1].

Among the potential drug handling changes in cirrhosis are the reduced hepatic blood flow, lower first-pass extraction, and portosystemic shunting that determine a higher bioavailability and serum levels for most drugs [3,5,58]. Drugs with an ordinarily high rate of liver first-pass extraction will typically have reduced bioavailability. However, due to decreased hepatic blood flow and presence of porto-systemic shunts, these drugs have higher bioavailability and serum concentrations often require dose reductions [5].

The presence of portosystemic shunts, including TIPS, may lead to increased bioavailability of some drugs. This could be a problem for some drugs that can prolong the QTc interval, especially considering that cirrhotics patients have baseline QTc interval prolongation (due to the portosystemic shunting of cardioactive substances derived from the splanchnic circulation) [144]. Among antibiotics, fluoroquinolones may prolong QTc intervals in patients with TIPS [145], and appropriate care should be taken. 

The macrolide antibiotics erythromycin and clarithromycin are also known to prolong the QTc interval; in their review, Vuppalanchi et al., observed that after a 7-day course of erythromycin, cirrhotic patients with a TIPS developed a significantly more significant prolongation in their QTc interval (180 ± 68 ms) compared with both the cirrhotic patients without TIPS (31 ± 10 ms) and with the healthy controls (38 ± 3 ms) (*p* = 0.03) [145]. These findings should prompt caution when prescribing medications known to prolong QTc in cirrhotic patients who have undergone a TIPS procedure, especially those patients who are being prescribed a fluoroquinolone for SBP treatment or prophylaxis [5].

## 6. Conclusions

In conclusion, bacterial infections are one of the most frequent complications and a significant cause of death in cirrhotic patients, and therefore it is crucial to know how to use antibiotics correctly. The safe use of medications in patients with chronic liver disease is an ongoing challenge, and this is especially true in patients with cirrhosis, in whom significant challenges can occur in the metabolism and handling of a large number of drugs. In general, in order to decide drug dosing in liver failure, three essential factors need to be considered (1) pharmacokinetic alterations of drugs, (2) pharmacodynamic alteration of drugs, and (3) increased susceptibility of patients to adverse events, particularly hepatotoxicity. In patients with cirrhosis, the drug dosing should be individualized depending on several factors, including renal function and nutritional status, adherence, and drug interaction [1].

In general, most drugs can be used safely in cirrhosis, including potentially hepatotoxic ones, but lower doses or reduced dosing frequency are often recommended due to altered pharmacokinetics. Drugs that can precipitate renal failure, gastrointestinal bleeding, SBP, and encephalopathy should be identified and avoided [5].

Moreover, whenever possible, measuring drug levels in the blood and monitoring adverse events frequently should be done. Early diagnosis and appropriate antibiotic treatment are the first steps in the management of cirrhotic patients with infection. While third-generation cephalosporins remain the gold-standard strategy in most community-acquired infections, the empirical treatment of nosocomial and healthcare-associated infections should be adapted to the local epidemiological pattern of antibiotic resistance [146] often requires the use of broad-spectrum antibiotics, such as piperacillin-tazobactam, carbapenems or tigecycline. Moreover, prompt de-escalation of antibiotic treatment is recommended to prevent the development of antibiotic resistance [14].

We have attempted to summarize current knowledge and translate it into relevant and practical recommendations through this narrative review. However, the current and increasing spread of MDR bacteria, especially in the nosocomial setting, represents a significant challenge in the correct management of cirrhotic patients.

## Figures and Tables

**Table 1:** Antibiotic use in ascites and SPB.

Antibiotic Use in Ascites and SPB	
**Community- acquired SPB** First line therapy of SPB: Cefotaxime 2 g/8 h IV or Ceftriaxone 2 g/24 h IV or Amoxicillin-clavulanate 1–0.2 g/6–8 h IV Other options: Ciprofloxacin 400 mg/12 h IV or Ofloxacin 400 mg/12 h PO (in uncomplicated SBP) **Nosocomial SPB** - Meropenem 1 g/8 h IV * - Daptomycin (i.e., 8–12 mg/kg per 24 h) plus meropenem (i.e., 1 g/8 h) ** - Tigecycline 100 mg IV loading dose followed by 50 mg/12 h IV **	
**Recommendation in hepatic impairment**	
**Cefotaxime**	No need for dose adjustment (wide drug therapeutic index)
**Amoxicillin/Clavulanate**	No need for dose adjustment (renal tubular secretion)
**Ciprofloxacin**	No need for dose adjustment
**Tigecycline (nosocomial SPB)**	Mild to moderate hepatic insufficiency: no need for dose adjustment Severe hepatic insufficiency: dose should be reduced by 50%
**Carbapenems (nosocomial SPB)**	No need for dose adjustment

* Areas with a high prevalence of ESBL producing *Enterobacteriaceae*. ** Clinical setting with a high prevalence of VRE, MRSA, ESBL.

**Table 2:** Antibiotic use in hepatic encephalopathy.

Antibiotic Use in Hepatic Encephalopathy		
Antibiotic	Spectrum of Activity and Mechanism of Action	Dosage
Rifaximin	Active against Gram-positive, Gram-negative, and anaerobic enteric bacteria Binds DNA-dependent RNA polymerase and disrupts RNA synthesis	**550 mg orally every 12 h** **or 400 mg orally every 8 h** Better results if coupled with lactulose
Neomycin	Active against most Gram-negative aerobes, except some pseudomonas strains, and against *S. aureus* and *E. faecalis*. Inhibits bacterial protein synthesis via binding to the bacterial 30S ribosomal subunit, causing misreading and premature termination of mRNA translation	**Acute episode: 1 g orally every 6 h for up to six days** **Chronic use: 1–2 g orally daily**
Metronidazole	Active against anaerobic bacteria, protozoa, and microaerophilic bacteria Inhibits protein synthesis by interacting with DNA and causing helical DNA structure and strand breakage loss	**No more recommended to manage the acute episode or chronic management of HE**
Vancomycin	Active against Gram-positive bacteria, including MRSA: effective for *Streptococci*, *Enterococci*, and methicillin-susceptible *Staphylococcus aureus* (MSSA) infections Inhibits the polymerization of peptidoglycans in the bacterial cell wall	
